# Challenges of implementing the integrated disease surveillance and response strategy in Zambia: a health worker perspective

**DOI:** 10.1186/s12889-017-4791-9

**Published:** 2017-09-26

**Authors:** Chomba Brian Mandyata, Linda Kampata Olowski, Wilbroad Mutale

**Affiliations:** University of Zambia School of Public Health, Lusaka, Zambia

## Abstract

**Background:**

Despite advances in medical technology and public health practice at the global level over the past millennia, infectious diseases are still the leading causes of death in most resource limited countries. Stronger infectious disease surveillance and response systems in developed countries facilitated the near elimination of infectious disease related deaths in those countries. Today, low-income countries are following this path by strengthening disease surveillance and response strategies that would help reverse the trend in infectious disease associated morbidity and mortality cases. In 2000, Zambia adopted the World Health Organisation Regional Office for Africa’s (WHO-AFRO) Integrated Disease Surveillance and Response Strategy (IDSR) to monitor, prevent and control priority notifiable infectious diseases in the country. Through this strategy, activities pertaining to disease surveillance are coordinated and streamlined to take advantage of similar surveillance functions, skills, resources and targeted populations. The purpose of the study was to investigate and report on the existing challenges in the implementation of the IDSR strategy in a resource limited country from a health worker perspective.

**Methods:**

A qualitative study approach was used to achieve the study aim. Data was collected through key informant interviews with selected persons at the Lusaka Province Health Office (LPHO); Lusaka and Chongwe District Health Management Team Offices; and four selected health facilities in the two districts (two from each). Thematic analysis approach was used to analyse the qualitative data.

**Results:**

The major successes included operationalised response and epidemic preparedness at all levels (National to district); full-time staff and budget dedicated to disease surveillance at all levels and adoption of the 2010 World Health Organisations’ Integrated Disease Surveillance and Response Strategy technical guidelines to the Zambian context. Several challenges hampered effective implementation. These include inadequate trained human resources, poor infrastructure and coordination challenges.

**Conclusion:**

The implementation of IDSR strategy in Zambia has recorded some successes. However, several gaps hinder effective implementation. It is imperative that these gaps are addressed for Zambia to have a robust surveillance system that could inform policy in a comprehensive and timely manner.

## Background

A disease surveillance system that continuously and systematically collects, analyses, interprets and utilise health data for decision making at an optimum level is a corner stone of an effective public health system [1, 2]. Disease surveillance systems provide information about disease manifestations and severity, etiological characteristics of the disease, their space-time distributions, the use of and potency of treatments that is vaccines and so on and so on [3–5].

During the 1990s, most African health systems extensively implemented vertical disease surveillance and response strategies for each priority infectious disease that was targeted for control and/or elimination. Several drawbacks had been identified with these types of systems and these included: high cost of maintaining the various parallel systems; inability of the several vertical disease surveillance strategies to adequately fulfil the functions of surveillance and response; heavily centralised systems; inability to detect disease outbreaks in a timely manner; duplication of work due to lack of coordination between several single disease control and prevention programmes; overburdened health personnel responsible for disease surveillance in terms of workload and so on [6–11]. Furthermore, these vertical disease surveillance strategies were also failing to cope with the increasing ease of travel of their targeted populace (mostly propagated by air travel), the rapid urbanisation of African cities, and the associated public health challenges that come with them coupled with the incremental threat of emerging and re-emerging diseases of pandemic potential alongside endemic diseases such as Human Immunodeficiency Virus (HIV), Hepatitis and other diseases. Meanwhile, the financial costs for implementing these vertical programmes kept on skyrocketing while at the same time most African economies at the time were either declining or remained stagnant.

This situation in the continent of Africa at that time prompted the World Health Organisation Regional Office for Africa (WHO-AFRO) to develop a cost effective and efficient disease surveillance and response strategy for African member countries. The strategy was adopted under resolution AFR/RC48/R2 by the WHO-AFRO member countries in September 1998 when the World Health Organisation Regional Committee for Africa met in Harare, Zimbabwe [12].Some of the aims of the IDSR strategy are to: “train personnel at all levels; develop and carry out plans of action; advocate and mobilise resources; integrate multiple surveillance systems so that forms, personnel and resources can be used more efficiently; improve the use of information to detect changes in time to conduct a rapid response to suspected epidemics and outbreaks; monitor the impact of interventions; facilitate evidence-based response to public health events; and inform health policy design, planning and programme management; improve the flow of surveillance information between and within [various] levels of the health system; strengthen laboratory capacity and involvement in confirmation of pathogens and monitoring of drug sensitivity; emphasise community participation in detection and response to public health problems including event based surveillance and response in line with IHRs [International Health Regulations of 2005]” [12].

Under article 5.1 of the resolutions of the IHRs, it is stated that each country will have to develop, strengthen and maintain, as soon as possible but no later than five years from the date of entry into force of the resolutions for that particular country (June 2007 for Zambia) the capacity to detect, assess, notify and report public health events of international concern in accordance with the set parameters contained within the resolutions [13]. These regulations require that each member country develops, operates and manages a real time health event monitoring and strengthened surveillance system [14].

In Zambia, the IDSR has been used to complement the Health Management Information System (HMIS) in reporting detected priority notifiable infectious diseases to the relevant authorities within the Ministry of Health [15]. Within the HMIS, there are indicators for 11 priority notifiable infectious diseases which are reported to the next level in the reporting chain immediately they are detected/suspected and/or confirmed and these include: Acute Flaccid Paralysis (AFP); Measles; Neonatal Tetanus; Dysentery; Cholera; Plague; Rabies; Typhoid Fever; Yellow Fever; Tuberculosis (TB) and Human Influenza [15]. Notifications of these diseases and health events to the public health authorities in Zambia is mandated by law under the Public Health Act of 1995 [16], Ministry of Health regulations that is, the 2011 Technical Guidelines on IDSR in Zambia [17] and by the IHRs of 2005 [13].

Surveillance data collection is conducted mainly at the health facility level where in most cases paper-based information systems are used to collect information about suspected and confirmed priority notifiable infectious diseases and the associated mortality cases. Tallied information from these tools is then sent to respective District Health Management Team Offices (DHMTs), who then feed the validated data into the District Health Information System version II (DHIS II) – an internet based system with the main aim of reducing the reporting burden in primary health care settings by focusing and easily making available essential information for district level planning [18].

### IDSR implementation structure in Zambia

**Fig. 1:**
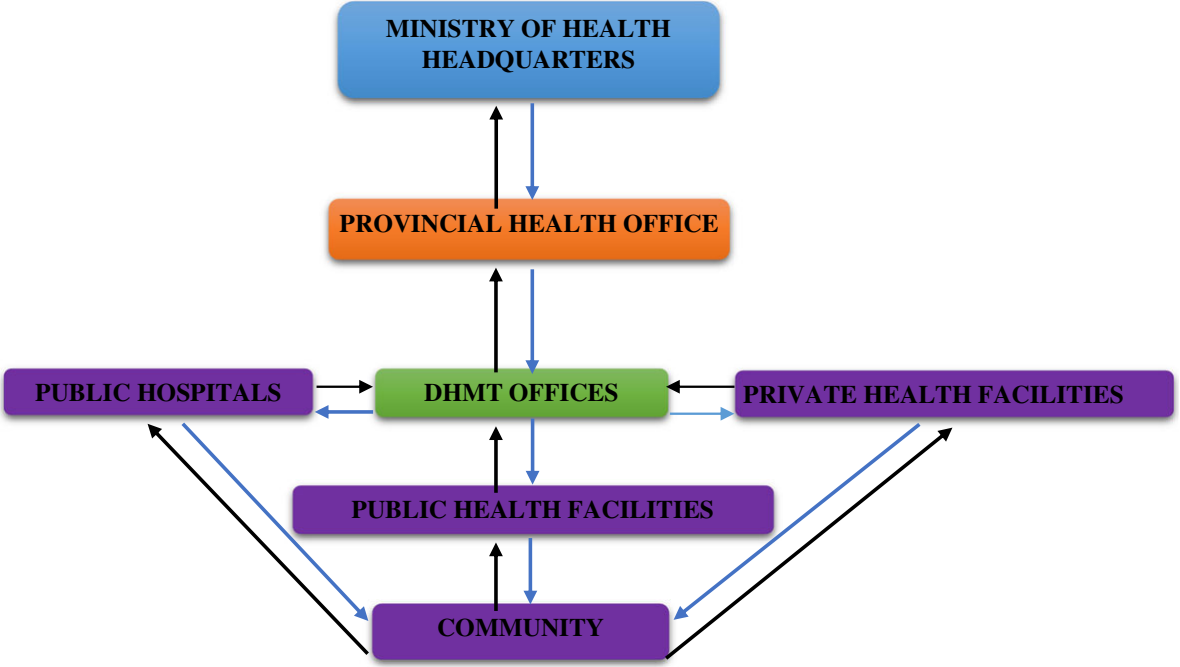
IDSR Implementation Structure

In order to effectively and efficiently achieve the aims of the IDSR in the Zambian public health system, the Ministry of Health developed and operationalised the IDSR implementation structure. It emanates from the community level up to the national level. Figure 1 below further illustrates this structure.

It shows the surveillance data flow from the community level up to the Ministry of Health headquarters. When members of the community suspect a disease, it is expected of them to report themselves and/or others to the nearest health facility. In the event that the health facility detects/suspects a notifiable infectious disease(s), it is required of them (health facilities) to report such cases to their respective District Health Management Teams (DHMTs) within a specified period of time usually on a weekly and monthly basis. Once the DHMTs receive the surveillance data, the health information unit through the District Health Information Officer (DHIO) then compile, validate, analyse and disseminate the received surveillance counts to other office units that is, policy and planning, environmental health, health promotion and so on within the respective DHMT offices. The disease surveillance unit at the DHMT institutes and leads further epidemiological investigations into any suspected and confirmed priority notifiable infectious disease and/or any public health event of concern with technical support from the respective Provincial Health Offices. At the same time, the DHMTs forward the received surveillance counts to the Disease Surveillance Unit at the Provincial Health Office who perform the same processes on the received data as the DHMTs. Once everything has been deemed to be satisfactory (by approval of the Provincial Disease Surveillance Officer), the respective Provincial Health Offices then send the provincial surveillance counts to the Ministry of Health headquarters. The disease surveillance section at the Provincial Health Office is mandated to provide supervisory and technical support to the DHMTs under their jurisdiction in all disease surveillance activities including case investigations and response. The monthly disease surveillance counts are typically compiled and managed by the Monitoring and Evaluation unit mostly by the District Health Information Officers (DHIOs) while weekly disease surveillance counts are compiled and managed by the Epidemiological section of the Ministry of Health through the Disease Surveillance Officers – where these positions have been filled. Otherwise, DHIOs or the Environment Health Officers (EHO) also perform the duties of a Disease Surveillance Officer. The aim of the study was to investigate and report on some of the existing challenges in the implementation of the Integrated Disease Surveillance and Response Strategy in a low-income country such as Zambia by documenting the health worker perspectives.

## Methods

### Study setting

Geographically, Lusaka province is centrally located on the map of Zambia. It covers a total surface area of approximately 21, 896 km^2^ with an estimated total population of 2, 191, 225 [19]. In the east, the province borders Mozambique at Luangwa district and Zimbabwe in the south at Chirundu district. The province has a total of seven districts namely; Lusaka (provincial and country administration capital), Chirundu, Chilanga, Chongwe, Kafue, Luangwa and Rufunsa.

### Study design

The study utilised a qualitative approach in its quest to achieve the study aims. Primary qualitative data was collected through key informant interviews with purposively sampled health workers at all levels of IDSR implementation.

### Sampling procedure

**Fig. 2:**
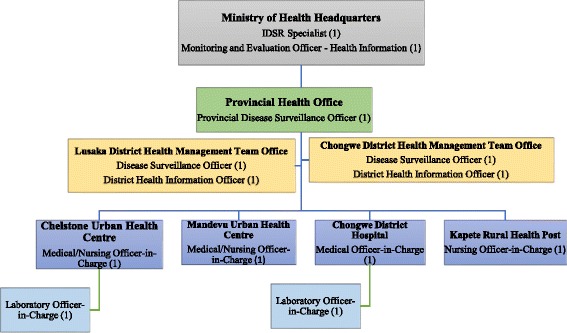
Flow Chart of Sampled Health Workers and their Positions in the Work Hierarchy

Figure 2 above shows the hierarchy (within the IDSR implementation structure) of key informants that were interviewed for this study. The study had purposively sampled the Ministry of Health headquarters and Lusaka Provincial Health Office (LPHO). The study then conveniently sampled two district health administration offices (one urban and one rural) both of which are under the jurisdiction of the LPHO and these were; the Lusaka District Health Management Team Office (LDHMT) located in an urban area; and the Chongwe District Health Management Team Office (CDHMT) – a rural district (Chongwe) located about 40 km east of Lusaka district. In each of the two sampled districts, two health facilities were purposively sampled. At least one of these health facilities in each sampled district had to possess an in-house laboratory capacity of some kind. All health facilities sampled were under the direct supervision of their respective DHMTs.

The sampling of only two districts is adequate to show the status of the IDSR implementation for all the other districts and health facilities in the country. This is because the procedures for implementing the IDSR is standardised for all districts and facilities (public or private) irrespective of their size, status or location that is urban or rural, health post or district hospital. This standardisation is stipulated in the 2011 Technical Guidelines for IDSR in Zambia [17] and the Public Health Act of 1995 [16]. Therefore, the findings from this study are transferable to other similar districts throughout the country.

### Sampling of key informants

Targeted key informants were those that were directly involved in the implementation of the IDSR at each level of health service delivery. From the Epidemiological Unit – which falls under the Directorate of Public Health, Disease Surveillance and Research, an IDSR specialist responsible for overseeing the optimal implementation of the IDSR strategy at the national level was interviewed. From the Directorate for Policy and Planning, a Monitoring and Evaluation (M&E) Officer was interviewed. The M&E officer is responsible for health information and management of all monthly health indicators (including those concerning infectious diseases that are covered by IDSR) that are submitted through the DHIS II by all District Health Management Team Offices country wide. At the provincial level, the study had sampled one key informant from the disease surveillance unit which is responsible for all disease surveillance activities in the province as well as receiving and compiling weekly IDSR reports from all districts under its jurisdiction. This unit is responsible for instituting and leading disease outbreak investigation efforts in the province. These responsibilities are the same for the district surveillance unit – though restricted to within district boundaries. At each of the two sampled DHMTs, two key informants were sampled; one officer from the health information unit; and the other from the disease surveillance unit. The health information unit is responsible for the collection, management, analysis and dissemination of health data on both communicable and non-communicable diseases as well as on risk behaviours that are of public health concern within the district. The health information unit is also responsible for receiving and compiling monthly reports on selected notifiable infectious diseases and other indicators ranging from service delivery to drug usage at health facilities under their jurisdiction in the district.

At the sampled health facilities with an in-house laboratory, two key informants were purposively sampled; the Laboratory Officer-in-Charge and the Medical/Nursing Officer-in-Charge. The Laboratory Officer In-Charge is responsible for all laboratory related activities at the health facility and for entering information about detected diseases in the laboratory register as well as on a weekly and monthly basis to compile and submit reports on tested and/or detected priority notifiable infectious diseases at the health facility to the Medical Officer/Nursing in – Charge. Coupled with the day to day administration of the health facility, the Medical/Nursing Officer-in-Charge is responsible for compiling and submitting weekly and monthly reports on suspected, confirmed and mortality cases on priority notifiable infectious diseases seen at the health facility to their respective DHMTs. All in all, a total of thirteen health workers that were eligible and consented to participle in this study were interviewed.

### Data collection

Data collection was conducted between January and March 2016. Interview guides were used in collecting data from the selected key informants. The study had four separate but related interview guides for each of the selected key informants. These interviews guides were for the following key informants: I) national, provincial and district surveillance officers; II) national and district information officers; III) Medical/Nursing Officers-in-Charge; IV) Laboratory Officers-n-Charge. The questions in the interview guide were adapted from the World Health Organisation (WHO) Protocol for the Assessment of National Communicable Disease Surveillance and Response Systems [20] and the Communicable Disease Surveillance and Response Systems: Guide to Monitoring and Evaluating [21]. The interview guides were developed and administered by the main author. The duration of the interviews ranged between 30 and 60 min. Each interview was recorded on a digital recorder. The principal investigator also took notes during the interview process. At the end of each interview, a typed transcript was then developed from the audio of the interview.

### Data analysis

Thematic analysis approach was used to aid the data analysis process. This study utilised the deductive technique of qualitative data analysis [22]. This was done by predefining or identifying four major themes of the study. These themes were based upon the four components of the IDSR implementation strategy namely; structure; quality attributes; core functions and support functions [12, 21]. The sub-components of each of these four major components of the IDSR were treated as sub-themes of the study. The themes that were falling outside the predefined analysis criteria were labelled and categorised separately. The coding and analysis of the collected data was done by the main author with oversight from the co-authors.

### Ethical considerations

Ethical approval was necessary due to the fact that, the study involved human subjects and required asking them about their experiences. In-depth interview guides were used in this study, this raised the risk of the participants delving into personal and politically sensitive matters, hence the need to protect the study participants from these vulnerabilities by seeking ethical approval. Ethical approval for this study was obtained from the University of Zambia Biomedical Research Ethics Committee (UNZABREC) assurance No. FWA00000338 IRB00001131 of IORG0000774. Permission from the Permanent Secretary at the Ministry of Health (the chief administrator of the ministry) and the National Health Research Authority were obtained to conduct data collection within the Ministry. Informed consent was obtained from all participants prior to conducting the interview.

## Results

Given the fact that the IDSR strategy is broad as it covers a wide array of activities that are supposed to be effectively implemented to achieve the ultimate goal of timely infectious disease detection and prevention and due to limited time and space, in this study, the researchers purposively selected certain key areas from each of the four components of the IDSR strategy that the researchers felt would to some extent highlight some of the main challenges of implementing the IDSR strategy within the Zambian health system. While the researchers acknowledge the fact that the studied areas of the IDSR strategy in this paper may not be incredibly extensive, it is believed that the findings (based on the selected IDSR strategy implementation areas) do highlight some (not all) of the prevailing challenges in the implementation of IDSR strategy that are ultimately contributing to the high rates of morbidity and mortality cases associated with priority infectious diseases such as Typhoid Fever and Measles in Zambia. The selected key areas of implementation are presented in Table 1 below.

**Table 1:** Emerging Themes from Key Informant Interviews

Main themes	Sub-themes
Structure	Legal and regulatory framework
Core functions	Case detection
	Case confirmation
	Case registration
	Case reporting
	Surveillance data analysis
	Response and control
	Feedback
Support functions	Training
	Logistical (financial, material and human resource) support
	Monitoring and evaluation
	Supervision
Quality attributes	Representativeness
	System stability

### Legal and regulatory framework

IDSR implementation in Zambia is governed by the Public Health Act of 1995, the IDSR technical guidelines, and the International Health Regulations of 2005. Most participants felt that the Public Health Act of 1995 was adequate to govern the effective implementation of the IDSR in the province, although there was a general sentiment that the existing legal and regulatory frameworks were not adequately responding to the current IDSR implementation environment. One key informant had argued that the Public Health Act of 1995, in particular, was not properly aligned with the International Health Regulations of 2005 to which Zambia is a signatory. While the Act covers a broad area of notifiable infectious diseases, it was seen to be weak in providing a legal framework that would be necessary to govern the detection, management and prevention of emerging and re-emerging infectious diseases and events of public health concern that is, H1N1 virus, Zika virus, bioterrorism which are not specifically covered by the Act. The following are some of the perspectives key informants had offered with regard to whether the Public Health Act of 1995 in its current form was adequate enough to provide a legal environment that would bring about an effective and efficient implementation of the IDSR strategy:

*“… all issues of prevention, reporting of cases, events and conditions exist within the Public Health Act of 1995 specifically under the section for notifiable diseases and most of the notifiable diseases are the IDSR diseases, only that this time around decision (parameters) have been changed. When you look at the International Health Regulations of 1969 and the International Health Regulation of 2005, they are no longer mentioning that this disease or that disease, instead they are saying any case, condition or event that is unusual or is of international public health concern should be reported”. (*Key Informant MoH Headquarters)

*“I do not think they are because you cannot just have one regulation or document that is a guiding principle for the entire implementation of the IDSR. If you look at the Technical Guidelines for the IDSR, you will see that actually, they is a lot that is involved and may be if we can have back up of some other laws, then it will be easier”.* (Key informant LPHO)

### Core functions

#### Case detection

The study findings revealed that at the LPHO, the log of rumours and suspected outbreaks (used to track the time taken between the first-time rumours and/or suspected outbreaks were recorded and the time action was taken) was non-existent, instead, they relied more on the notification reports. When asked if they have a log of suspected outbreaks, events and rumours, one participant at district level had this to say:

*“A log, we do not have, but we only have reports of rumours investigated, outbreaks investigated and so on. Any rumour that we hear we always investigate/follow ups”.* (Key Informant – DHMT)

Our findings also revealed that none of the four (4) health facilities that were visited in Chongwe and Lusaka districts had copies of the Zambian Technical guidelines on IDSR, although most of them had copies of the Standard Operating Procedures. The Technical guidelines on IDSR do provide stipulations on the procedures of handling suspected cases of a priority notifiable infectious disease at the facility level. Availability of these guidelines especially at the clinical level and their effective implementation at that level is the foundation of a strong disease surveillance system particularly in the early detection of priority notifiable infectious diseases and events of public health concern. However, what this study has found is that currently there is a challenge in ensuring that the simple procedures of that is, recording and investigating any rumour of a suspected disease or events of public health concern, promptly recording, reporting and obtaining laboratory confirmation of any suspected priority notifiable infectious disease, and optimal utilisation of the IDSR technical guidelines at all levels of IDSR implementation was inconsistently being done.

### Case confirmation

Our findings revealed that the two laboratories that were visited had the capacity to test for notifiable infectious diseases such as; Dysentery, Malaria, HIV and Tuberculosis (TB) or those diseases that can be ascertained by simple serological tests. For those diseases that require more advanced laboratory techniques such as culturing, whenever they are suspected, samples have to be collected and sent to the few existing referral laboratories dotted around the country with the largest one being the central laboratory at the University Teaching Hospital in Lusaka. Cooler boxes are used to transport the collected samples to the referral laboratories. What our study results revealed was that, there is a time delay in most lower health facilities that is, urban and rural health centres between the time a priority notifiable infectious disease such Typhoid Fever is suspected and the time it is confirmed at the referral laboratories (and communicated back to the health facility that sent the samples) and the time appropriate treatment is instituted on the affected patients. And this is attributable to the suboptimal laboratory capacities at most district hospitals as well as urban and rural health centres to confirm diseases that require culturing techniques and the fact that the referral laboratories where some of these tests can be done are usually hundreds of kilometres away.

In terms of water supply, both laboratories had consistent supplies; each health facility had at least one borehole as a water source coupled with supplies from the Lusaka Water and Sewerage Company. This study further found that only the T – lymphocyte cell bearing CD4 receptor (CD4) machines were connected to the backup power generators at both laboratories. The study also found that the supplies of reagents and other laboratory materials from Medical Stores was relatively consistent although they would be some months when supplies would be erratic especially when the suppliers did not have the materials that have been requested for. Supply of new laboratory stock is also dependent on monthly reports submitted to Medical Stores. One key informant had the following to say on the consistency of the central Medical Stores in providing the necessary materials to the laboratories at the visited health facilities:

*“…not very good because at times you find that some of the things we ordered if they do not have they don’t supply. But for HIV test kits they are very consistent… At times, they could be one or two or three months when they could be challenges with the supply. Basically, what you report is what you get. The supply chain is report dependent. The supply of laboratory material is dependent on the report”. (*Key Informant - Chongwe health facility)

### Case registration

In terms of registration of every case that is seen at the health facility, the study found that in some health facilities particularly those with a high patient demand clinicians are failing to comprehensively enter the appropriate information in the tally sheets, disease aggregation forms and other patient information collection documents available within their offices of operation. One of their arguments as one of the participants (Key Informant DHMT) put it is that: *“I see a lot of patients, tallying [of cases seen on each day] will delay my work”*. Key informants also indicated that the situation was also similar in those health facilities which at most times have low patient demand, thus clinicians have much more time on their hands. However, even in these kinds of health facilities (ones with low average daily patient demand) clinicians simply are not willing to consistently and completely enter and tally information about the cases that they come across at their respective health facilities on each particular day they are on duty*.* We further found that, in order to work around this challenge of not tallying complete information about cases seen, some health facilities have been engaging data clerks who on a weekly and monthly basis go through each of the patient’s books, disease aggregation forms, patient and laboratory register entries and/or other patient documents to extract information to be reported to the respective DHMT by Monday or the first working day of the following week for the weekly IDSR reports and by the 7th of the following month for the monthly surveillance reports on priority notifiable infectious diseases.

It was also found that even where they are data clerks available to extract the priority notifiable infectious disease surveillance data from the various patient documents and registers, the illegibility of most clinicians’ handwriting is proving to be a barrier to their ability to extract correct information. In some instances, the actual diagnosis as determined by the clinician may not be clear, hence in such situations, the data clerks then have to look at the prescription to determine and sometimes guess the actual diagnosis, due to the illegibility of the attending clinician hand writing. Thus, even when surveillance counts are sent to the respective DHMT on a weekly and monthly basis, the counts may not be the actual representation of the cases seen for that particular period (reporting week or month):

*“This means that data is missing, and it is missing because the clinicians are overwhelmed [by the high patient demand] and they have no time to tally all the cases that they see. Equally, the clerks are also overwhelmed because of the huge number of patient books and other materials from which they are supposed to uplift data from and make a weekly and monthly report. So, at the end of the day, they just do what they feel they should do”.* (Key Informant – DHMT)

### Case reporting

Once the weekly number of suspected and confirmed cases seen at the particular health facility have been tallied, they are entered in the standardised reporting forms provided by the respective DHMT offices. Health facility laboratories were available also make reports on the number of samples they have sent to the referral laboratories within a particular week. In instances whereby they are more than average numbers of cases that are being seen at a particular time, a line list is also used to collect information about the cases that are being attended to and these are sent together with weekly and/or monthly surveillance reports. Note that, the DHMTs only receives reports from health facilities under their jurisdiction and the largest facility at the district level is the district hospital – a level one hospital. General, central and teaching hospitals are not supervised by the DHMTs within the district where they are located but are supervised by the Ministry of Health (MoH). Although, these larger hospitals are expected to report any suspected, confirmed and mortality cases associated with priority notifiable infectious cases to the DHMTs from where the disease was originating from (i.e. patient resides in Ndola district in the Copperbelt province but was diagnosed in Lusaka district in Lusaka Province) they usually do not unless the designated district surveillance officer requests for the information. Once, the DHMTs receive the weekly reports from the respective health facilities and upon cleaning the data sent, they also tally the surveillance data received and submit a weekly IDSR report to the Provincial Disease Surveillance Officer at the Provincial Health Office (PHO). In most cases, when the DHMTs are sending weekly IDSR reports to the PHO they also attach copies of notification reports (which highlight preliminary background information about the affected patient[s]) which are compiled by health facilities. However, what this study found is although these notification reports are much more detailed than the IDSR reports, they are not treated as disease surveillance reports themselves. Only the aggregated information in the weekly IDSR reports is treated as disease surveillance data. The information they provide (notification reports) is only used to aid the suspected notifiable infectious disease outbreak investigations. Note that the IDSR reports submitted to the DHMTs, PHOs and MoH headquarters only highlight total counts of suspected, confirmed and mortality cases seen in that particular week. Key variables such as age, gender, the area of residence, date of first attendance, types of samples collected are not included in the reports. The variables found within the notification where they are reported according to a key informant at the Lusaka Provincial Health Office include such things as:

*“Age, gender, place of residence, occupation, date of first attendance, phone numbers, next of kin, specimen that were taken, whether or not they were confirmed, the actual diagnosis among other things. It also contains the historical background for that particular patient and whether or not the patient had died and what was done after that, recommendations and conclusion are also provided.”* (Key Informant – LPHO).

Note that, the information that is contained within the notification reports is not the information that is entered in the Excel worksheets (treated as databases) at the DHMTs and PHOs. Only information that is contained in the weekly IDSR reports is entered in the Microsoft Excel work sheets. The other challenge we found was that (at the time of the study), the weekly IDSR reports had not yet been fully incorporated in the DHIS II for reporting to the next level. This is despite the fact that, the Ministry of Health rolled out the DHIS platform as far back as 2007 and around 2012, the Ministry upgraded the system to DHIS II. As a result, weekly reports are sent to the next level through phone calls, email and sometimes through the delivery of hard copies on a weekly basis:

*“The [weekly] surveillance data is not sent through the DHIS II. The disease surveillance unit have their own database [Microsoft Excel Worksheets] – created by the surveillance unit. They compile a weekly report and submit it through email on a weekly basis. For those who are unable to email, they have hard copies that are blank which they fill in on a weekly basis. ”* (Key Informant – LPHO).

This study also found that there is a parallel and well-established reporting structure for the monthly notifiable infectious disease surveillance reports which are sent to the M&E unit (under the Directorate for Policy and Planning) through the use of the DHIS II. This system is available currently at the district level, however, it is not yet available at the health facility level. On a monthly basis, health facilities tally all information about suspected and admitted cases of all notifiable infectious diseases as well as their associated mortalities that they had seen during that month. This information has to be submitted to the DHMT by the 7th day of every month. Once the information has been validated at the district level, the DHIO now enters this information in the DHIS II which makes the information instantaneously available to anybody who has access to the system. This information should be entered in the system by the 21st of every month. Thus, there is a 14-day delay between the time DHMTs receive monthly surveillance counts from the respective health facilities and the time this information is entered in the DHIS II:

*“Before the data is even entered …, you check through the facility reports. If you find that there are issues you can even retain the report to the facilities for them to read through. Then it can be resent. But of course, the person who is sending the data may not be able to check through every indicator. So, certain indicators, you will find that they are okay while in others they may be some lapses…”* (Key informant – DHMT)

### Surveillance data analysis

Our study findings revealed that the weekly IDSR reporting form does not have the person (that is, age and gender) and place (that is, residential area) variables, only aggregate figures are provided in the report. The findings showed that the main form of analysis conducted is through the construction of trend lines and/or disease monitoring charts as recommended by MoH (see [23]). Each reporting surveillance officer either from the DHMTs reporting to the Provincial Health Offices or this reporting to MoH headquarters gives a brief analysis and discussion of the figures that they had received in the previous week and/or month. When asked whether or not weekly trend and disease monitoring charts, as well as trend lines, were being consistently constructed one key informant had the following to say:

*“…we do that, but on a quarterly basis but it’s not like every day or every week but from our data, we are able to see that Measles, for example, is coming down or it’s going up. Once we see that it is going up or down we notify the next level*. *”* (Key informant – LDHMT).

Microsoft Excel is used to tally and analyse the received weekly IDSR reports while in most cases the statistical functions available in the DHIS II are normally used to analyse the monthly disease surveillance reports. Advanced statistical software such as Stata, SPSS and so on are used only in times when they need to do some further digging on the data.

Surveillance data has to be analysed by person and time as well as by place. One of the most accurate ways to analyse surveillance data by place is through the utilisation of the Geographical Information System (GIS). However, currently our findings revealed that this tool (GIS) is not being utilised in aiding the accurate understanding of the precise geographical distribution of priority notifiable infectious diseases in the country:

*“We used to have what is called the health mapper, [for] GIS… what you should bear in mind is that we do not have a system now that is in a sharp we would have loved it too. But when we had EPI info system, mapping was provided, meaning that you can do (analyse) your data and show it. Even at this (national) level, we were able to analyse and show which district and in which province or which province has a particular disease. If we wanted to particularise to a district we would be able to paint the districts that are affected. If we wanted to show which health facilities within the particular district where the cases were coming from, we were able to show those health facilities.” (*Key informant - MoH Headquarters)

### Response and control

The study findings revealed that at the provincial and district levels, the Rapid Response Teams (RRTs) have been created and includes such specialised officers such as the: Disease Surveillance Officers, clinical care experts, nursing officers, environmental health officers, transport unit, and laboratory unit:

*“…as a province, we have a Rapid Response Team [RRT]. This RRT will first do an on-spot check of the data that was sent. For example, if it is Typhoid Fever or Cholera that has been reported, we will go there as a team to investigate and verify what they [DHMTs] have sent. Then if they is need to support them materially, then we do that. But usually what is there is that we have logistics and supplies that are set aside for such things. So, if they [DHMTs] need any further support from the provincial health office that is, financially or materially then we come in to help.”* (Key informant – LPHO).

### Feedback

Validated and analysed disease surveillance counts on specific priority notifiable infectious diseases is disseminated (feedback) back to the lower levels of the implementation hierarchy as highlighted by the arrows pointing downwards in Fig. 1 above. Feedback is provided through quarterly or annual reports, statistical bulletins, supervisory visits, newsletters, workshops and seminars. However, this study found that feedback to the lower implementation levels was not being done in a consistent manner – that is, the Provincial Health Offices sending feedback to respective DHMTs and from these to the health facilities and then finally to the communities. Participants indicated that feedback is at most times provided when the senders have done something wrong that is the presence of errors in the report, have sent higher or lower than usual numbers of suspected and/or confirmed priority cases or during the times of a disease outbreak:

*“It is usually when there is something wrong that is when you get that feedback. And also, when you have a meeting and you present your data that is when you will hear some comments on your data. But not immediately that somebody views your data, and gives you feedback. ”* (Key Informant DHMT).

*“[with regard to us] sending data [feedback] to the health facilities we have not been doing that, but we are supposed to do it. But what we do normally is that when we see some strange disease trend from some of our reporting facilities, we call them – we notify them. ”* (Key Informant DHMT).

### Support functions

#### Training

Key informants especially those at the periphery levels revealed that they have not yet been trained in IDSR although they have a primary role in the implementation of the strategy within their respective districts. The main reason that was given was that these trainings are expensive and at most times there is usually no funding specifically for training in IDSR. In instances where health workers are trained in most cases, it is just an orientation to the system especially for the newly recruited health staff:

*“…remember this thing came with donor funding – but what is there now is that where we see gaps we just do an on-site orientation. For example, if we see that a particular DHMT is not doing fine in terms of reporting we do an onsite orientation there and then just to impart knowledge on the IDSR.” (*Key informant - LPHO)

### Logistical support

In terms of logistical support, we found that transportation facilities, particularly at district and facility levels, was the major challenge. At the district level, the unit responsible for district surveillance in most cases has to rely on pool vehicles to conduct its activities as they do not have their own transport facilities. At the facility level, the challenge is even deeper. Due to the general lack of transports facilities, health workers in some cases have to use their own initiative in order to transport samples to referral laboratories for disease confirmations – sometimes at their own costs. Where they can, the core implementers (Ministry of Health Headquarters and Provincial Health Offices) do provide logistical support to the respective DHMTs and their respective health facilities:

*…transportation is one of the biggest challenges affecting our work here at the district. If we as a unit can have our own transport instead of relying on pool vehicles [it] would make our work much easier*. (Key Informant DHMT)

### Supervisory visits, monitoring and evaluation

Our study findings revealed that supervisory visits were not being done in a regular manner and that it is usually only in times of disease outbreaks that is when supervisory visits to the periphery levels are done. One of the main reasons cited was the lack of funding from Central Government for such activities. Furthermore, a clinician interviewed revealed that supervision would at times be conducted when they (clinical staff) visited their respective District Health Management Team offices:

*“Supervisory activities are not done due to funding. For 2015 only one was done [at a provincial level.”* (Key informant – LPHO).

### Quality attributes

#### Representativeness of IDSR surveillance data

The findings from this study have revealed that so far most of the weekly and monthly IDSR data that is reported to the DHMTs is mostly from the public health facilities. DHMTs are still struggling to get the private health facilities to submit the weekly and monthly IDSR reports despite several attempts requesting them to send reports regardless of whether or not they have had a case of a priority notifiable infectious disease:

*“Majority of the health institutions that submit the weekly reports are the public health centres. However, we are still struggling to incorporate the private health facilities, we have had meetings with these institutions but for them to send data here they are finding it a problem. But for a few like Lusaka Trust Hospital whenever they have a case that is notifiable, they call, they have my number and we go there and collect information and then we disseminate to the relevant authorities.”* (Key Informant – DHMT).

### IDSR system stability

Stability here refers to the duration and consistency of operation of the system [24]. This study also tried to gauge the stability of certain aspects of the IDSR system by asking the key informants to give an estimate on the frequency of internet outage that they experience in a specified period of time – in this case, six months (this is relevant as a bulk of communication between the different IDSR implementation levels is done via the internet). Most of the key informants had indicated that they experience internet outage when; power supply to their offices has been cut mostly due to load-shedding; subscription fees to the service providers have not been paid by the respective health offices; and at times even when there is internet connectivity, it often is so slow that officers cannot download or upload files either through their emails addresses or through the DHIS II in a timely manner. In order to ensure that reports are sent on time, most officers at the periphery as well as at the core of the IDSR system resort to the use of their personal internet access mostly through their mobile phones at their own cost:

*“In most cases, there is internet only when they is power, however, we are heavily load-shaded here at the office. Hence, in most of the cases, we have to rely on our own internet mostly through mobile phones … for districts the situation is quite bad. Since most of them depend on their grants to pay for such services as internet connectivity … at the moment, grants are a bit erratic, there isn’t much funding from the central government. Worse even at the centre level, for they just use their own initiative to send these reports”.* (Key Informant – LPHO)

## Discussion

The study has shown that the Ministry of Health has made significant strides in the adaptation and implementation of the IDSR strategy to the Zambian context. The strength of the system is that it has been rolled out to all health facilities throughout the country. The technical guidelines for IDSR in Zambia make it explicit that all health facilities public or private have to report all suspected, and confirmed mortality cases associated with any of the priority notifiable infectious diseases stipulated within the guidelines and the Public Health Act of 1995. The guidelines even go further by requiring all health facilities to submit weekly and monthly reports on selected priority notifiable infectious diseases regardless of whether or not they have had a case. The Ministry has also established an IDSR implementation structure with clearly defined roles and responsibilities for each position from national to facility level. There is also a dedicated budget plan for IDSR implementation which is revised on a regular basis. However, despite these strengths, they are still gaps that are hampering the optimal implementation of the strategy. On the core functions side of the strategy, the ministry is still facing challenges in the effective detection, registration and reporting of cases to the higher levels. While these challenges emanate from a multi-facet of sources, health workers’ attitude, inadequate and ill-trained human resources (in IDSR), high patient demand, several reporting requirements, inadequate availability of necessary materials and tools, and poor information and communication technology infrastructure are directly contributing to the dismal performance of the system [7, 25, 26].

Health worker motivation, especially at district and facility levels, was particularly negatively impacted by the inadequacy and inconsistency feedback that is provided to the lower levels. Health workers are not adequately informed on their performance concerning the tallying and submission of weekly and monthly disease surveillance counts and how their efforts are contributing to the fight against priority notifiable infectious diseases in the community where they work. The study has also shown that it is not only the lack of feedback that is affecting the optimal performance of the system in detecting, preventing and controlling notifiable infectious diseases but also the health workers lack lustre attitude towards recording, tallying and reporting of all cases that they see at their respective health facilities. While the poor enforcement of the Public Health Act, technical guidelines on IDSR and other regulations are some of the contributors to this negative attitude, the heavy leaning of the general health system in Zambia towards curative medicine at the expense of preventive medicine through public health and the high patient to medical personnel ratio are other contributing factors. Weaknesses in providing appropriate technical support especially transportation and communication facilities are also significantly contributing to the inability of the health workers particularly at district and facility levels to adequately carry out their assigned IDSR implementation duties. These facts were found to be re-enforcing the sub-optimal performance of the other areas of the core functions that is, case registration, reporting, analysis and response, and control. Consistent feedback coupled with other incentives (that is improved technical support) and disincentives for defaulters was found to significantly contribute to improvements in the quality, timeliness and completeness of reporting of monthly and weekly disease surveillance reporting in Peru and Tanzania [25, 27].

Sub-optimal performance of the core function side of the strategy was also re-enforced by poor implementation of the support side of the strategy [28, 29]. Training of key front line staff on IDSR was still inadequately being done. At the same time, the technical guidelines on IDSR implementation in Zambia [17] are also not readily made available particularly at health facility level. Health workers mostly rely on their experiences and academic backgrounds in order to execute their duties with regard to IDSR - which may not be adequate as disease surveillance is not specifically offered as a course in medical colleges and universities in the country. This is further having an impact on the quality and quantity of the disease surveillance data that is being generated, transmitted and utilised for decision making in the Zambian health system. The higher the number of key frontline staff trained in IDSR, the higher the reported improvements in the quality of reporting, feedback, supervision, monitoring and evaluation including timeliness and completeness of reporting in the health systems of Cape Verde, Eritrea, Ethiopia, Guinea Bissau, Tanzania, South Sudan, Gambia, Uganda and Malawi [25, 30, 31]. Competent disease surveillance staff at all levels of health service delivery are a necessity especially in a resource limited country like Zambia for rational planning, implementation and infectious disease prevention and control [32].

These weaknesses coupled with other broader health system gaps that is the inadequate enforcement of the Public Health Act of 1995 [16] and other local and international regulations on health service delivery in Zambia, health financing, inadequate human resources, logistical and technical support and so on., are all reflected in the sub-optimal performance of the IDSR particularly on the quality attributes of timeliness and completeness of reporting as well as in the management of disease surveillance data at national level.

## Conclusion

The Ministry of Health has over the years made significant strides in the quest to have a system that would specifically be used to detect, prevent and control priority notifiable infectious diseases in the country in the most effective and efficient manner. So far, the Ministry has put in place an IDSR implementation structure with clearly defined goals and measurable indicators. The ministry has also created dedicated disease surveillance positions, epidemic preparedness committees, and rapid response teams from national to district levels. However, a number of gaps still remain. These include inadequately trained human resources, lack of provision of optimal technical support to the DHMTs and health facilities, poor infrastructure and coordination challenges. For as long as these challenges remain unattended to, the number of preventable morbidity and mortality cases associated with priority notifiable infectious diseases in Zambia will continue to be high. It is, therefore, of utmost importance that the Ministry of Health comprehensively addresses the challenges that have been raised in this study in order to improve decision making within the health system and to inform policy and ultimately, to effectively and efficiently detect, prevent and control priority notifiable infectious diseases in Zambia.

## Abbreviations

AFP: Acute Flaccid Paralysis
CDHMT: Chongwe District Health Management Team
DHIO: District Health Information Officer
DHIS II: District Health Information System Version II
DHMT: District Health Management Team
EHO: Environment Health Officer
EVD: Ebola Virus Disease
HIV: Human Immunodeficiency Virus
HMIS: Health Management Information System
IDSR: Integrated Disease Surveillance and Response
IHR: International Health Regulations
LDHM T: Lusaka District Health Management Team
LPHO: Lusaka Provincial Health Office
M&E: Monitoring and Evaluation
MOH: Ministry of Health
PHO: Provincial Health Office
TB: Tuberculosis
UNZABREC: University of Zambia Biomedical Research Ethics Committee
WHO – AFRO: World Health Organisation Regional Office for Africa
WHO: World Health Organisation
